# MedT5SQL: a transformers-based large language model for text-to-SQL conversion in the healthcare domain

**DOI:** 10.3389/fdata.2024.1371680

**Published:** 2024-06-26

**Authors:** Alaa Marshan, Anwar Nais Almutairi, Athina Ioannou, David Bell, Asmat Monaghan, Mahir Arzoky

**Affiliations:** ^1^School of Computer Science and Electronic Engineering, University of Surrey, Guildford, United Kingdom; ^2^College of Business Studies, PAAET, Kuwait City, Kuwait; ^3^Surrey Business School, University of Surrey, Guildford, United Kingdom; ^4^Department of Computer Science, Brunel University London, London, United Kingdom; ^5^School of Business and Management, Royal Holloway, University of London, London, United Kingdom; ^6^Department of Computer Science, Brunel University London, London, United Kingdom

**Keywords:** text-to-SQL conversion, large language model, transformers, T5 model, NLP, MIMICSQL dataset, healthcare domain

## Abstract

**Introduction:**

In response to the increasing prevalence of electronic medical records (EMRs) stored in databases, healthcare staff are encountering difficulties retrieving these records due to their limited technical expertise in database operations. As these records are crucial for delivering appropriate medical care, there is a need for an accessible method for healthcare staff to access EMRs.

**Methods:**

To address this, natural language processing (NLP) for Text-to-SQL has emerged as a solution, enabling non-technical users to generate SQL queries using natural language text. This research assesses existing work on Text-to-SQL conversion and proposes the MedT5SQL model specifically designed for EMR retrieval. The proposed model utilizes the Text-to-Text Transfer Transformer (T5) model, a Large Language Model (LLM) commonly used in various text-based NLP tasks. The model is fine-tuned on the MIMICSQL dataset, the first Text-to-SQL dataset for the healthcare domain. Performance evaluation involves benchmarking the MedT5SQL model on two optimizers, varying numbers of training epochs, and using two datasets, MIMICSQL and WikiSQL.

**Results:**

For MIMICSQL dataset, the model demonstrates considerable effectiveness in generating question-SQL pairs achieving accuracy of 80.63%, 98.937%, and 90% for exact match accuracy matrix, approximate string-matching, and manual evaluation, respectively. When testing the performance of the model on WikiSQL dataset, the model demonstrates efficiency in generating SQL queries, with an accuracy of 44.2% on WikiSQL and 94.26% for approximate string-matching.

**Discussion:**

Results indicate improved performance with increased training epochs. This work highlights the potential of fine-tuned T5 model to convert medical-related questions written in natural language to Structured Query Language (SQL) in healthcare domain, providing a foundation for future research in this area.

## 1 Introduction

Large businesses, government departments, healthcare providers, financial services and many others store their vast amounts of data in large relational databases or [data centers. To handle, manage and retrieve information from these databases, it is required to know the necessary technical background which non-technical people lack. For example, Structured Query Language (SQL), a standardized programming language that performs a variety of data operations to manage databases, provides special communication with databases typically required for efficient data management, including retrieval, deletion and updating records (Groff et al., 2002). One prominent use of relational databases is in today's healthcare domain, where patients' health information is stored in databases as electronic medical records (EMRs), designed to ensure that every patient receives the correct medical care, based on their entire health history. EMRs also help researchers gather the statistics required for clinical trials, in turn helping the study of diseases and the provision of suitable cures. To carry out their duties, healthcare professionals must be able to access EMRs, however, while they are considered experts in their medical fields, they often lack formal training in database query languages like SQL. This can result in significant inefficiencies when attempting to extract relevant patient information from Electronic Medical Records (EMRs). Studies have shown that clinicians spend a considerable amount of their time on documentation and data entry tasks, often leading to frustration and burnout (Shanafelt et al., 2012; Sinsky et al., 2016). A survey of over 4,000 physicians revealed that 49% reported spending more than half their workday interacting with EHRs (American Medical Association, 2018). Moreover, the complexity of EMR databases, with their intricate schemas and vast amounts of data, can further exacerbate these challenges. This difficulty in accessing data can hinder clinical decision-making, delay patient care, and impede research efforts. For instance, a study found that difficulties in retrieving relevant information from EMRs contributed to diagnostic errors in 25% of cases (Singh et al., 2013). Therefore, an intermediate system is therefore needed that can assist end-users, such as the healthcare staff, to handle database records smoothly without needing to learn SQL.

**Figure 1:**
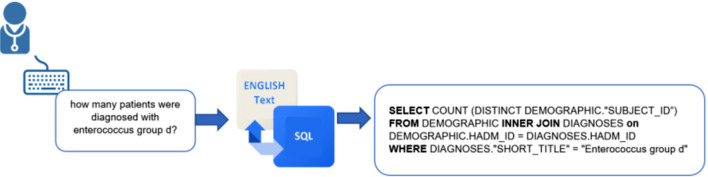
Text-to-SQL conversion model.

Responding to this need, researchers started to explore the possibility of employing automated Text-to-SQL conversion, using machine learning (ML) and natural language processing (NLP) to convert questions written in natural language to SQL queries; the principle is shown in Figure 1 (Iyer et al., 2017; Kate et al., 2018; Kim et al., 2020). NLP is a pervasive artificial intelligence (AI) technology in which computers simulate human intelligence through machine learning. Without explicit programming, machine learning automates the learning of computers using a collected data based on the required task. In this way, computers are given the ability to understand human language and turn it into machine language to perform required tasks, such as text summarization and translation. Text-to-SQL conversion facilitates the development of flexible, highly interactive communication with databases to handle the records without the need for end-users to know SQL.

Previous research papers have analyzed the creation of SQL through NLP and proposed Text-to-SQL conversion models such as SQLNet, proposed by Xu et al. (2017), Seq2SQL, developed by Zhong et al. (2017) and MedTS, created by Pan et al. (2021). Recently, the NLP technology has progressed with the development of Transformer, a deep neural network architecture capable of multiple NLP tasks, such as automatic summarization and translation (Vaswani et al., 2017). This architecture became the baseline for various language models trained on large data to perform NLP tasks, such as Bidirectional Encoder Representations from Transformers (BERT), proposed by Devlin et al. (2019) and Multi-Task Deep Neural Networks (MT-DNN) for Natural Language, proposed by Liu X. et al. (2019). Transfer learning these pre-trained models, in which they are fine-tuned on a downstream task such as translation, has become an effective approach in NLP research. In Text-to-SQL conversion, fine-tunning pre-trained models has raised the performance of Text-to-SQL models to near human performance levels (Guo et al., 2019; Wang et al., 2019; Pan et al., 2021). Subsequently, Raffel et al. (2020) proposed their model, namely Text-to-Text Transfer Transformer (T5) as a unified model for various NLP tasks and is considered one of the first Large language Models (LLMs). The T5 model transforms text-based language problems, such as translation, into a text-to-text format and has become the state-of-the-art for various NLP tasks, such as summarization, question answering and text classification (Raffel et al., 2020; Xie et al., 2022). Using the T5 model for Text-to-SQL conversion resulted in a significant improvement in the performance of such task (Scholak et al., 2021; Xie et al., 2022).

While many researchers have proposed Text-to-SQL conversion models, few have focused explicitly on the healthcare domain to assist healthcare staff in managing and retrieving information from EMRs (Wang et al., 2020; Pan et al., 2021). This relative scarcity can be attributed to several factors. First, healthcare data presents unique challenges, including complex medical terminologies, diverse data formats across different EMR systems, and stringent privacy and security requirements. These challenges necessitate the development of specialized Text-to-SQL models that can accurately understand medical language and comply with healthcare-specific regulations. Second, the integration of Text-to-SQL systems with existing EMR systems can be complex and time-consuming. The heterogeneity of EMR systems across different healthcare institutions, with varying data structures and terminologies, poses a significant barrier to generalizability. Developing a Text-to-SQL model that seamlessly integrates with diverse EMR systems requires extensive customization and validation, which may deter researchers and practitioners from focusing on this domain.

Despite these challenges, the need for efficient and user-friendly access to EMR data remains critical for healthcare professionals. Therefore, this work aims to develop a T5-based model, namely MedT5SQL, which is a transformers-based fine-tuned large language model to perform Text (questions)-to-SQL conversion specifically within the healthcare domain. The objective of the MedT5SQL model is to empower medical staff by enabling them to express their data requests in natural language, thereby overcoming the barriers associated with traditional SQL query formulation.

In achieving the above, this paper is structured as follows. First, a theoretical background of the work related to text-to-SQL conversion is discussed. The following section clarifies the research methodology, namely CRoss Industry Standard Process for Data mining (CRISP-DM), that is followed to pre-process the data and develop and validate MedT5SQL model. Third, the evaluation results are discussed in detail and compared to past research. Finally, the conclusion section concludes this work and offers some suggestions for future research.

## 2 Theoretical background

Nowadays, patients' health information is stored in a digital format in electronic medical records (EMRs) that are used by healthcare staff to retrieve patients' historical health details or to use for clinical trials. At the beginning of 2020, the world experienced a global pandemic of coronavirus known as COVID-19. This pandemic has left hospitals overloaded with patients, causing enormous stress on healthcare workers due to shortages of medical staff in relation to the number of patients (Birkmeyer et al., 2020; Kruizinga et al., 2021; Iness et al., 2022). The pandemic highlighted the importance of EMRs and revealed the need for a faster communication method to handle it (Dagliati et al., 2021). It is essential to have an interface that provides easy user-to-database interactions; in particular, a system that generates an SQL query in response to a question in human language. This section reviews the state-of-the-art in natural language processing for Text-to-SQL conversion to facilitate interactions between users and databases.

### 2.1 Rule-based systems

Converting natural language to SQL is a subtask of semantic parsing, in which natural language is converted into a machine-understandable logical form (Zettlemoyer and Collins, 2005). Semantic parsing seeks to understand the meaning of natural language and map it to logical forms such as SQL. Rule-based systems were used to support non-technical users in communicating with databases through a set of predefined rules mapping natural language words with SQL keywords and database schemas (Androutsopoulos et al., 1995; Popescu et al., 2004; Li and Jagadish, 2014; Saha et al., 2016). An expert programmer constructs these rules to translate users' requirements into SQL queries (Masri et al., 2019). However, it is required for non-technical users to train before using them and are domain-specific, since each system is built for a specific schema. These systems have limited intelligence, as they only operate based on the rules created by humans and do not learn, change or update on their own (Kamath and Das, 2018). This limits the ability of non-technical users to manage their data without relying on expert programmers.

### 2.2 Deep learning models for text-to-SQL

To increase usability and generalize Text-to-SQL conversion, researchers began using deep learning (DL) by training neural networks to generate executable SQL queries. Training neural networks means performing supervised learning, in which the network is provided with natural language questions and their corresponding SQL queries so it can learn the conversion. The trained neural networks is called a DL model that generates a query from a given question. This has led to the release of several Text-to-SQL datasets that boost the accuracy of the models by delivering sufficient data for supervised learning: GeoQuery, created by Zelle (1996) for US geography and updated later by Iyer et al. (2017) to include SQL; ATIS, created by Price (1990) for flight bookings and updated by Iyer et al. (2017) to include SQL; Scolar, created by Iyer et al. (2017) for academic publications; WikiSQL, created by Zhong et al. (2017) from Wikipedia; and Spider, created by Yu et al. (2018a) and representing a cross-domain dataset.

**Figure 2:**
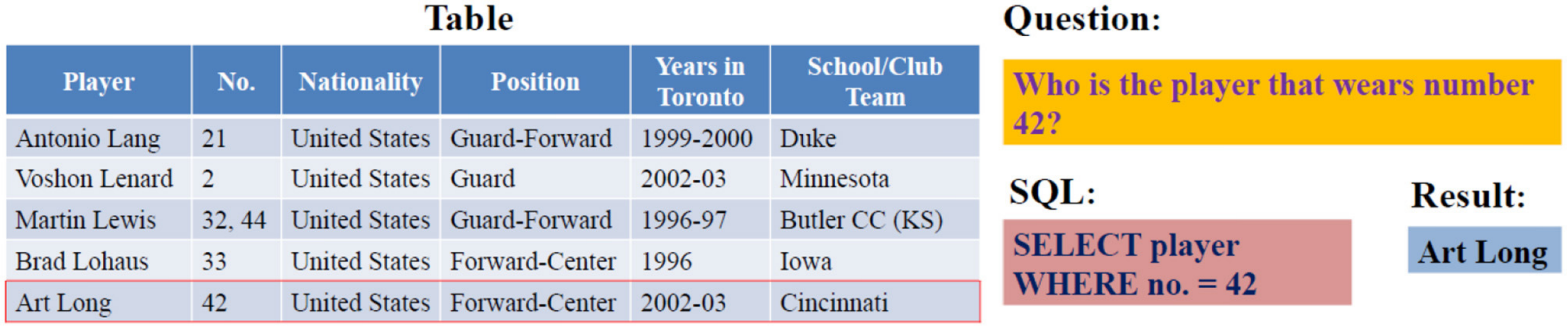
Sample of WikiSQL question-SQL pairs (Xu et al., 2017).

Due to their large sizes and multiple domain coverage, Spider and WikiSQL are the most used datasets among researchers. WikiSQL is a corpus of 80,654 hand-annotated pairs of questions and corresponding SQL queries for 24,241 tables covering multiple domains. However, each question-SQL pair is related to a single table in which the SQL only has SELECT and WHERE clauses, as presented in Figure 2. The Spider dataset was introduced to overcome WikiSQL's simple SQL structure and to present the first cross-domain dataset. It includes 200 complex databases with multiple tables, 10,181 questions, and 5,693 corresponding complex SQL queries with nested queries. Table 1 presents a comparison of existing datasets for text-to-SQL translation.

**Table 1:** Comparison of existing text-to-SQL benchmarking databases.

**Dataset**	**#Databases**	**#Tables per database**	**#Question-SQL pairs**	**SQL query level**
ATIS	1	32	5,280	Complex (no HAVING and ORDER BY)
GeoQuery	1	6	877	Complex
Scolar	1	7	817	Simple
Spider	200	On average 5	10,181	Complex
WikiSQL	24241	1	80,654	Simple

#### 2.2.1 Deep learning models architecture

Deep learning models for Text-to-SQL conversion are built as neural networks in an encoder-decoder architecture that was initially embraced by Sutskever et al. (2014) for translation purposes. Given a natural language question (NLQ) and its corresponding SQL as source sequences, models operate as follows:

- The source sequences are always tokenized into tokens before encoding, and each token represents a word in the sequence (Webster and Kit, 1992).
- As deep learning models only take numbers as inputs, each token is embedded into a vector representation, called word embedding, using embedding algorithms such as Glove or Word2vec (Mikolov et al., 2013; Pennington et al., 2014). This process reveals the relationship between tokens and reduces input dimensionality as tokens with similar meanings have similar vector representation.
- The encoder takes the NLQ tokens' embeddings and encodes their information/features into a vector named “hidden states.”
- For training purposes, the decoder takes the encoder's hidden states and the word embedding of the SQL tokens for the supervised training. The decoder is built and trained as a classifier to decode the hidden states into a target SQL query.
- For generalization to an unseen schema, the database schema is usually considered as an input to the models.

To provide an accurate conversion, models must develop an understanding of source sequences by understanding words' dependencies and memorizing previously gathered information. To meet this need, researchers have built encoders and decoders with recurrent neural networks (RNNs), particularly long short-term memory (LSTM) (Hochreiter and Schmidhuber, 1997). LSTM can remember long-term information and capture dependencies between sequence tokens. The understanding and encoding of each token depends on the previously seen token. Therefore, it can improve natural language understanding and help with translation tasks (Graves, 2013; Yin et al., 2017).

**Figure 3:**
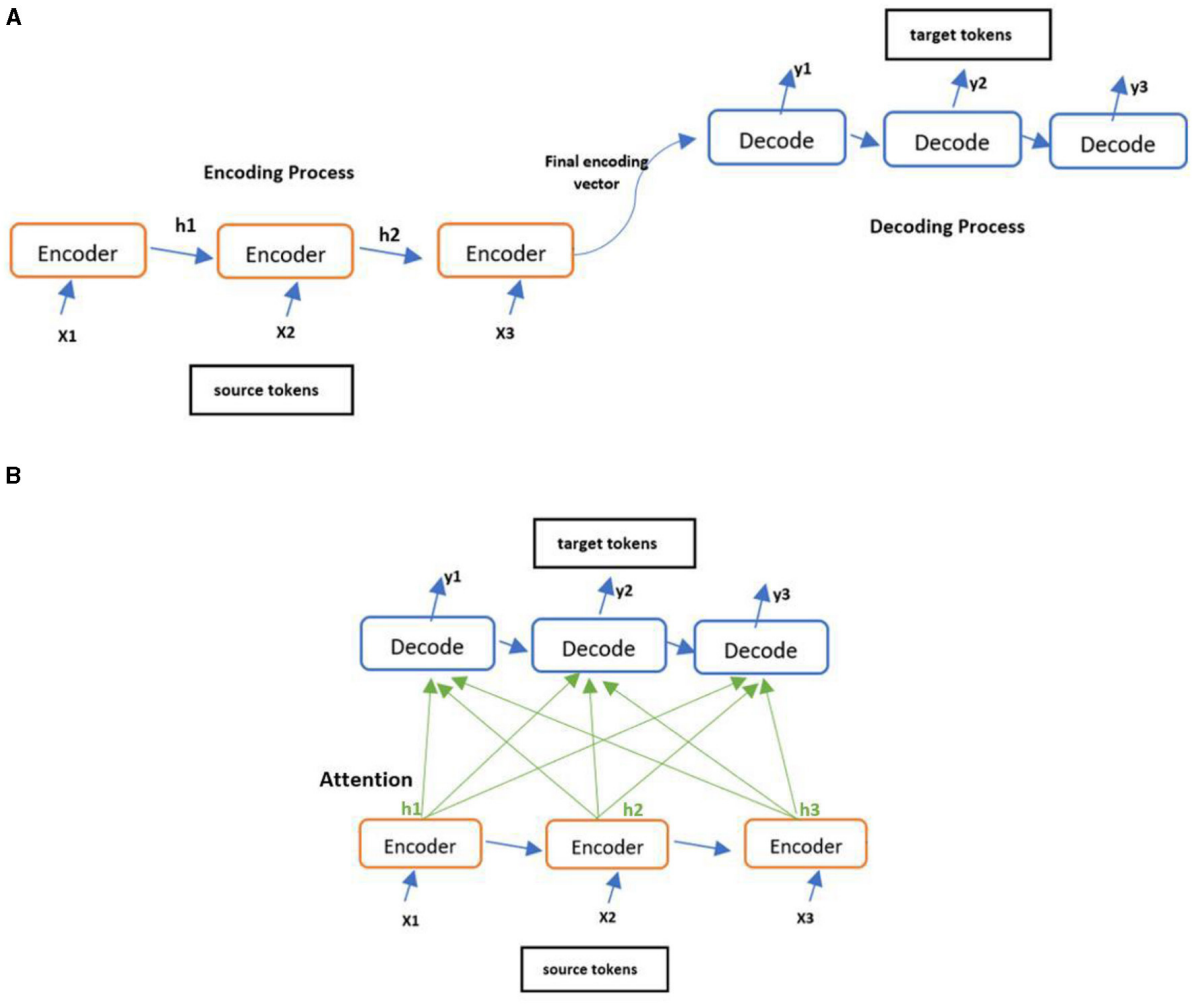
Various encode-decoder architectures. **(A)** Encoder-Decoder architecture and **(B)** Encoder-Decoder architecture with attention.

Encoders built using LSTM take input tokens sequentially and produce their hidden states one at a time. At the end, it outputs a single hidden states vector compressing all the tokens' hidden states. The decoder alone needs to interpret the information compressed in this vector into a complex target sequence, leading to the risk of information loss. To circumvent this risk, an attention mechanism was proposed to allow the decoder to look at all tokens' hidden states when predicting the final output (Bahdanau et al., 2014; Galassi et al., 2021). This is accomplished by passing the weighted sum of the hidden states to the decoder, allowing it to focus on the required information to generate the next target token. This simplifies the encoder task by avoiding encoding the entire source sequence into a single vector. The architecture of the encoder-decoder with and without attention mechanism can be seen in Figure 3 where ‘h' corresponding to hidden states vectors.

#### 2.2.2 Deep learning approaches

In Text-to-SQL tasks, this sub-section outlines the approaches used as (1) sequence-to-sequence (2) sequence-to-set (3) fine-tuning a pre-trained language model (transfer learning).

Sequence-to-sequence (Seq2Seq), introduced by Sutskever et al. (2014), is an LSTM-based machine translation that operates by sequentially taking source tokens and translating them into sequence target tokens. Seq2Seq relies on a single ground truth query as the optimal correct query. This raises the issue of “order matter” because in SQL, the order in the WHERE clause does not matter, making it a challenge when using this approach. Seq2Seq does not require the attention mechanism; however, it is possible to combine the two for better results.

Sequence-to-set was first introduced by Xu et al. (2017). It is similar to Seq2Seq, apart from its ability to overcome the order matter by producing an unordered set of sequences after dividing the prediction into sections. The decoder prediction is performed based on the dependency between the predicted tokens, which is captured using the attention mechanism. Sequence-to-set usually uses an approach of sketch matching and slot filling, where each slot has its own decoder. The slots present parts of the SQL, such as the column name or the aggregation operator, in the SELECT clause. Using a sketch structure presenting the dependencies of the query slots, the decoding of each slot in the query is based only on the decoding of other slots it depends on. For example, decoding the aggregation operator in the SELECT clause depends on the decoding of the column name and is independent of the WHERE clause.

Pre-trained language models are transformer-based neural networks for word embedding that learn contextual relations between tokens without recurrent connections (Peters et al., 2018; Yang et al., 2019). The Transformer is an encoder-decoder-based neural network proposed by Vaswani et al. (2017). It is built and trained to work on multiple NLP tasks, such as summarization and translation. The transformer has three main functioning concepts. The first is positional encoding, in which transformers are fed with all the tokens at once, with each token appended with its order, unlike the recurrent neural network of sequential input of token. Second, through learning from training data, transformers use the attention mechanism and consider each input token in the source before any translation prediction is generated. Third, both the encoder and decoder use a self-attention mechanism in which a word is understood based on the context of the words around it (Vaswani et al., 2017).

Although transformers are encoder-decoder neural networks, pre-trained language models only use the encoding mechanism, as they aim to learn representations of a language. The most commonly used language model in text-to-SQL conversion is bidirectional encoder representation from transformers (BERT), introduced by Devlin et al. (2019). The term “bidirectional” means positional encoding and the term “representation” refers to the attention mechanisms. BERT is a multi-layer bidirectional transformer encoder for contextual-bidirectional embeddings that can be finetuned for specific NLP tasks. It was trained by two learning mechanisms—masked learning mechanism (MLM) and next sentence prediction (NSP)—to increase its accuracy and minimize the loss values. In MLM, 15% of input tokens are placed with masked tokens (MASK) before being given to BERT. Therefore, through contextual relations between tokens, BERT learns to predict the original token. In NSP, BERT is given pairs of sentences and trained to predict whether the pairs are subsequent to each other in the source text. It is fed by 50% subsequent pairs during training, where sentences are separated by special tokens at the start of the first sentence in each pair and at the end of each sentence. Most pre-trained models were built later, based on BERT (Sanh et al., 2019; Liu X. et al., 2019).

To apply transfer learning with pre-trained models, researchers must perform fine-tuning by re-training the model using one of the Text-to-SQL datasets. In GloVe and Word2Vec, each token is embedded into one static vector representation. However, as a result of the attention mechanism in BERT, a token appearing in multiple locations in the source is treated as different tokens, thus embedded into multiple word embeddings/vectors based on its context.

Most of the text-to-SQL models were evaluated using:

*Execution accuracy:* this metric compares the results of executing the ground truth query (gold standard) with the results of executing the model-generated query. While intuitive, it can be misleading, especially in situations where multiple queries produce the same result. For instance, consider a query to find the average age of patients. Both SELECT AVG(age) FROM patients and SELECT AVG(age), COUNT(^*^) FROM patients would yield the same average age, but only the first query accurately captures the intent of the natural language question.

*Logical form accuracy (exact match):* This metric compares the structure of the ground truth query with the generated query using an accuracy matrix. It addresses the limitation of execution accuracy by focusing on structural correctness. However, it can be overly strict, as minor variations in query formulation (e.g., different ordering of clauses) can lead to incorrect results even if the queries are functionally equivalent.

*Manual matching:* In this approach, human evaluators manually compare the structure of the ground truth query with the generated query, often using a set of predefined criteria. Manual matching offers a nuanced assessment of query correctness, but it can be time-consuming and subjective.

*Combination of metrics:* Given the limitations of individual metrics, using a combination of execution accuracy, logical form accuracy, and manual matching provides a more comprehensive evaluation. Execution accuracy verifies the functional correctness of the query, while logical form accuracy and manual matching assess its structural correctness and alignment with the natural language question's intent.

#### 2.2.3 Text-to-SQL in single domain dataset

WikiSQL is considered the biggest single dimension dataset used for Text-to-SQL, where each SQL is related to a single database table. Seq2SQL, created by Zhong et al. (2017), was the first model trained with WikiSQL. It uses a Seq2Seq approach designed to leverage the structure of SQL commands with three decoders for the SELECT column clause, aggregation operator and WHERE clause separately. It uses two encoders, one for the question tokens and another for the column name, to train the model to generate the SQL query given the question and column. The decoder was designed as an LSTM-augmented pointer network created by Vinyals et al. (2015). It augments the encoder's output along with an SQL vocabulary of required SQL operations to produce the SQL query with tokens taken exclusively from this augmentation. To minimize the effect of the order matter problem, Seq2SQL uses reinforcement learning with policy gradients presented by Sutton et al. (1999), allowing the decoder to evaluate the predicted query based on whether it is well formed or not. The model achieved an execution accuracy of 59.4% and a logical form of 48.3%. Even though it presented a state-of-the-art model for WikiSQL, an accuracy below 50% is considered insufficient.

**Figure 4:**
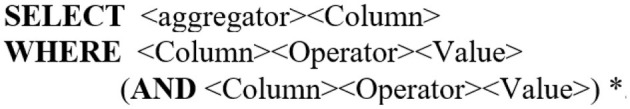
SQLNet SQL sketch.

SQLNet is a sequence-to-set sketch-based approach developed by Xu et al. (2017) to avoid the order matter. The dependency between the slots is based on the SQL sketch shown in Figure 4, where five decoders were used. Tokens between “ <>” are the slots to be filled, while (^*^) indicates zero or more conditions. The aggregator options are NULL, MAX, MIN, COUNT, SUM and AVG, while the operator options are =, > and <. Additionally, SQLNet uses a column-attention mechanism in which one LSTM encoder is used over each column name and another is used to encode the NLQ conditional in each column. In this way, the model reflects the most relevant word in the question when predicting the column name. SQLNet structure allowed it to achieve around 10% improvement in the execution accuracy compared to Seq2SQL. TYPESQL, developed by Yu et al. (2018c), is an improved version of SQLNet with a 5.5% increase in accuracy. TYPESQL achieves 2% higher accuracy by concatenating each NLQ token with a type before encoding to assist the decoder in filling the slots. For example, the model uses INTEGER, FLOAT, DATE or YEAR for number tokens, COLUMN for column name tokens and PERSON, PLACE, COUNTRY, ORGANIZATION and SPORT for named entities. TYPESQL achieves the other 3.5% by grouping related slots together, resulting in three decoders. All models use GloVe word embedding for the encoder embedding layer.

Due to their functionality, pre-trained models are effective in revealing the connections between source sequences as well as portraying the meaning of the question. Therefore, researchers began using them to connect questions with table schema to produce accurate SQL queries. Hwang et al. (2019) developed SQLova, the first model to utilize BERT in text-to-SQL tasks for word embedding on WikiSQL. SQLova was created following a sequence-to-set approach with LSTM. It has two separate encoders: one for the question and one for the column names. BERT is used on top of the encoders to perform word embedding for the question and column names. This allows the model to capture a larger context of the input with any possible different pronunciations of the question. Inspired by SQLNet, SQLova follows the same decoding process as well as a sixth decoder for “where-number,” indicating the number of conditions. SQLova uses the execution guided (EG) decoding proposed by Wang et al. (2018) to exclude non-executable generated queries from the decoder output. By using BERT, SQLova achieves 80.7% logical form accuracy and 86.2% execution accuracy without EG and 83.6% and 89.6% with EG. Therefore, using a pre-trained model increased the accuracy of this task.

X-SQL, created by He et al. (2019), was built based on the SQLova structure. Similar to SQLova, X-SQL uses two encoders for the question and table schema. However, the question encoder is built using the multi-task deep neural network (MT-DNN), a pre-trained model proposed by Liu X. et al. (2019) based on BERT. With and without EG, X-SQL outperforms SQLova by 2–4%. This implies that using a pre-trained model as an encoder rather than a word embedder results in better performance. Lyu et al. (2020) argued that neither SQLova nor X-SQL benefit correctly from using a pre-trained language model and added complexity to the models. They proposed Hydranet, employing BERT alone as its encoder without using any other encoders. Instead of pairing the question with all table schemas, Hydranet pairs the question with each column one at a time before encoding. Hydranet was able to achieve state-of-the-art on WikiSQL by reaching 91.8% execution accuracy using EG and 92.2% when replacing BERT with RoBERTa (Liu Y. et al., 2019). Even though those models keep increasing accuracy for text-to-SQL, they are trained on WikiSQL, which means they can manage simple SQL structures.

#### 2.2.4 Text-to-SQL in cross domain dataset

To develop text-to-SQL tasks for complex SQL queries, the Spider dataset was proposed, motivating researchers to develop models for more realistic SQL tasks. Yu et al. (2018c) evaluated SQLNet and TYPESQL on Spider to study their functionality for complex queries. It was found that both models failed to manage nested queries because they limited the query to a defined sketch structure. Motivated by SQLNet, they proposed SyntaxSQLNet (Yu et al., 2018b). As Spider question-SQL pairs can relate to multiple tables, SyntaxSQLNet encoding considers both tables and column names for column embeddings. They employed grammar-based decoding, in which a series of grammar rules are applied sequentially to generate the SQL query. By recursively calling nine independent sequence-to-set decoders, they obtained their SQL syntax tree to generate the SQL. In SyntaxSQLNet, decoders share their decoding history to facilitate the prediction of nested queries; thus, given the current training sample's SQL tokens and the history of previous decoded SQL, the relevant decoder is invoked. Even though its performance was better than SQLNet and TYPESQL on Spider, it achieved an accuracy below 30% due to the complexity of Spider's SQL.

Lee (2019) presented RCSQL, a clause-wise SQL decoding model, to predict syntactically correct SQL. Each clause decoder consists of sub-models matching its clause syntax and implied history sharing. For further improvement, they conducted a self-attention mechanism on database schema encoding. RCSQL's exact matching accuracy was 28.8%, indicating that improvement is still needed. IRNet, created by Guo et al. (2019), adopted the grammar-based model of SyntaxSQLNet. It focused on addressing the challenge of out-of-domain words affecting column prediction. They proposed the use of schema linking, where the model identifies the dataset's columns, tables and conditions appearances in the question. This enhanced the question and schema representations, aiding in their understanding. The model achieved around 20% improvement over SyntaxSQLNet. Inspired by SQLova, Guo et al. (2019) augmented BERT with both SyntaxSQLNet and IRNet. As a result, the performance of both models increased by around 5%. Choi et al. (2021) proposed a complete sketch to synthesize nested queries in the SELECT clause. They also proposed statement position code (SPC) to transform nested SQL queries into non-nested SELECT clauses and to apply sketch-based slot-filling decoding recursively on each statement. With BERT as an encoder, their model RYANSQL achieved 58.2% exact match accuracy on the Spider benchmark.

Unlike models built on WikiSQL, which deals with table schema, Spider models need to handle table schema relations or database schemas since the question-SQL pair represents multi-table relations. Accordingly, the researchers began contextualizing the dataset schema with the question to boost performance. As seen in IRNet, th performance improved with schema linking. RAT-SQL is a grammar-based model presented by Wang et al. (2019) with an encoder that contextualizes the schema and the question using a relation-aware self-attention mechanism. According to their alignment and schema relations, RAT-SQL explicitly links columns with corresponding question tokens, achieving logical form accuracy of 57.2% on Spider and 65.6% when augmenting with BERT. In BRIDGE, created by Lin et al. (2020), the relational DB schema is represented as a tagged sequence concatenated to the question. Using the database content, the model accesses the values of the columns identified in the question and appends them to their column names in the question. As a result, the input is a hybrid question-schema serialization containing the question, followed by the table name, column names, and column values. BRIDGE uses BERT to shape dependencies in the serialization and two single-layer LSTM encoders with a single LSTM-based pointer-generator with attention for decoding. This allowed the model to exceed the RAT-SQL by 1.9%. When applied to WikiSQL, BRIDGE was able to achieve 86.5% with EG.

#### 2.2.5 Text-to-SQL in healthcare domain

Despite WikiSQL and Spider being multi-domain benchmarks, they lack sufficient suitable medical records. Therefore, Wang et al. (2020) proposed the first dataset for healthcare named MIMICSQL. It consists of five tables and 10,000 question-SQL pairs of real-world medical information. The syntax for SQL does not include nested queries, but includes multiple tables connected by the JOIN operation. The pairs are divided into template questions and natural language questions based on the collection method: machine-generated (template questions) or human-annotated. Along with the database, they released TREQS, a translate-edit model operating in two stages. Stage one involves translating a natural language question into SQL using a Seq2Seq model with attention, while stage two performs editing to the generated SQL using a look-up table. The look-up table contains the table's names, columns and keywords of each column to recover the exact information between the question and the schema. They also proposed a technique to ensure query execution by retrieving the condition values of the predicted SQL and matching them against the dataset. As they introduced the model with their dataset, their accuracy measurements were broken down based on the question-SQL pairs. They achieved 85.3% and 92.4% logical form accuracy and execution accuracy, respectively, for template questions and 55.6% and 65.4% for human-annotated questions. Pan et al. (2021) claimed that because TREQS is based on Seq2Seq, it did not consider SQL's intrinsic structure. To incorporate the results of IRNET, they proposed a model named MedTS, which applied schema linking and BERT as an encoder. MedTS adopts a grammar-based LSTM decoding strategy with designed grammar rules based on the MIMICSQL dataset. A logical form of 78.4% and execution accuracies of 89.9% were obtained by MedTS.

### 2.3 Text-to-text transfer transformer (T5)

Raffel et al. (2020) conducted a large-scale survey on existing transfer learning techniques in natural language processing, such as ELMO created by Peters et al. (2018) and BERT created by Devlin et al. (2019). After testing and refining several models in NLP, they created a Text-to-Text Transfer Transformer (T5) model built on insights from the survey. The T5 model is a pre-trained language model that uses the complete encoder-decoder architecture of the transformer (Vaswani et al., 2017). In addition, T5 uses layer normalization to stabilize the hidden state and reduce training time (Ba et al., 2016). It is a very large neural network that takes the source sequence tokens all at once and relies on self-attention alone to compute its source input and target output. The T5 model was created as a unified framework covering all NLP tasks, such as summarization and translation, by converting every language problem into a text-to-text format. Unlike other pre-trained models, this model takes the source sequence as input and produces a target text string rather than word embedding.

The T5 model has various sizes depending on the number of parameters used for building and training it, as summarized in Table 2. The model was trained with two learning methodologies, as follows:

- Unsupervised training, in which T5 was trained on the colossal clean crawled corpus (C4) created by Raffel et al. (2020). C4 is a huge clean dataset of English text collected from the web for pre-training the T5 model.
- Supervised training, in which T5 was fine-tuned for several NLP tasks by training it with labeled data for each task. T5 was pre-trained using the Adafactor optimizer created by Shazeer and Stern (2018) and cross-entropy loss function. The loss function is used to evaluate the model performance during training by comparing the generated result with the expected result to produce a loss value (Demirkaya et al., 2020). The optimizer is an algorithm used to update the model parameters to reduce the loss value, such as inputs' weight presenting the impact of an input on the model output.

**Table 2:** Summary of T5 model sizes.

**T5 Model**	**Model Size**
Small	60 million parameters
Base	220 million parameters
Large	770 million parameters
3b	3 billion parameters
11b	11 billion parameters

In Raffel et al.'s (2020) evaluation, the T5 model achieved promising results on many NLP benchmarks and was shown to be flexible for fine-tuning a variety of NLP problems. Its development has shown that deep learning approaches are moving toward reaching human-level accuracy in performing NLP tasks. Xie et al. (2022) proposed a large-scale multi-task learning framework using T5 and studied its performance in 21 NLP tasks, including Text-to-SQL. On many SQL benchmarks, such as Spider and WikiSQL, their study showed that the T5 model achieved near and above the state-of-the-art performance of these benchmarks.

Inspired by Raffel et al. (2020), researchers have started considering the T5 model to directly convert NLQ into SQL with simpler architecture. Shaw et al. (2020) showed by experiment that the T5 model without modification achieved promising results compared to previous models on Spider. They proposed NQG-T5, a hybrid model combining a grammar-based approach with the T5 model, achieving competitive results with the state-of-the-art model on the Spider dataset with a 70% exact match accuracy using the T5-3b. In a study conducted by Scholak et al. (2021), the T5 model was fine-tuned on Spider and augmented with an additional method called PICARD at decoding. PICARD was implemented to guarantee semantically correct SQL by rejecting invalid tokens at each decoding step. To match the generated SQL with the question, PICARD uses the table schema when evaluating SQL tokens. They concluded that the conversion was accelerated, and performance was improved using the T5 model. Their T5+PICARD model became the state-of-the-art on Spider with 71% exact match and 75% execution accuracy.

### 2.4 Summary

In summary, for improved accuracy, Text-to-SQL conversion models are developed by deep learning with encoder-decoder architecture. As pre-trained models were introduced, researchers began focusing on employing transfer learning for Text-to-SQL conversion, which led to near-human performance level. Furthermore, using pre-trained models instead of building models from scratch simplified the process of model development. Upon its introduction, the Text-to-Text transfer transformer (T5) captured the attention of researchers due to its encoder-decoder transformer architecture and its multi-task training covering various NLP tasks, such as summarization and question answering. Researchers started fine-tuning the T5 model for Text-to-SQL conversion, which significantly improved the performance, making it state-of-the-art. Table 3 presents a summary of the Text-to-SQL models discussed in this review where ACCLF, ACCEX and EG indicate logical form accuracy, execution accuracy, and execution-guided, respectively.

**Table 3:** Summary of text-to-SQL model.

**Research**	**Model specs**				
	**DL approach**	**Domain**	**Performance**	**Transfer learning (Yes/No)**	**Opportunity**
Seq2SQL (Zhong et al., 2017)	Seq2Seq	Single	ACCLF: 48.3% ACCEX: 59.4%	No	The first model on WikiSQL
SQLNet (Xu et al., 2017)	Sequence-to-set sketch-based	Single	ACCEX: 68.0%	No	Avoid the “Order-Matter
TYPESQL (Yu et al., 2018c)	Sequence-to-set sketch-based	Single	ACCEX: 73.5%	No	Improving SQLNet
SQLova (Hwang et al., 2019)	Sequence-to-set	Single	ACCLF: 80.7% ACCEX: 86.2% –with EG— ACCLF: 83.6% ACCEX: 89.6%	Yes (BERT)	Utilize BERT in Text-to-SQL
X-SQL (He et al., 2019)	Sequence-to-set	Single	ACCLF: 83.3% ACCEX: 88.7% –with EG— ACCLF: 86.0% ACCEX: 91.8%	Yes (MT-DNN)	Utilize MT-DNN in Text-to-SQL
Hydranet (Lyu et al., 2020)	Pre-trained language model	Single	–with EG– ACCLF: 86.0% ACCEX: 91.8%	Yes (BERT)	BERT alone as encoder
			–with EG– ACCLF: 86.5% ACCEX: 92.2%	Yes (RoBERTa)	RoBERTa alone as encoder
SyntaxSQLNet (Yu et al., 2018b)	Sequence-to-set grammar-based	Cross-domain	ACCLF: 27.2%	No	First Model on Spider
RCSQL Lee (2019)	Sequence-to-set self-attention mechanism	Cross-domain	ACCLF: 28.8%	No	clause-wise SQL decoding with attention mechanism
IRNet (Guo et al., 2019)	Sequence-to-setgrammar-based	Cross-domain	–without BERT— ACCLF: 46.7% –with BERT— ACCLF: 54.7%	Yes (BERT)	Handle out-of-domain words in columns prediction + schema linking
RYANSQL (Choi et al., 2021)	Pre-trained language model	Cross-domain	ACCLF: 58.2%	Yes (BERT)	Handle nested SELECT clause +BERT as encoder
RAT-SQL (Wang et al., 2019)	Grammar-based with Pre-trained language model	Cross-domain	–without BERT— ACCLF: 57.2% –with BERT— ACCLF: 65.6%	Yes (BERT)	propose relation-aware self-attention mechanism
BRIDGE (Lin et al., 2020)	Pre-trained language model	Single + cross-domain	–on Spider– ACCLF: 67.5% –on WikiSQL– ACCLF: 91.9%	Yes (BERT)	hybrid question-schema serialization
TREQS (Wang et al., 2020)	Seq2Seq	healthcare	ACCLF: 55.6% ACCEX: 65.4%	No	First Healthcare Domain Text-to-SQL model
MedTS (Pan et al., 2021)	Grammar-based with pre-trained language model	healthcare	ACCLF: 78.4% ACCEX: 89.9%	Yes (BERT)	Introduce transfer learning to healthcare domain
NQG-T5 (Shaw et al., 2020)	Transformer	Cross-domain (In this work, the focus is on Spider)	On Spider development set: -Using T5-base- ACCLF: 57.1% -Using T5-3b- ACCLF: 70%	Yes (T5)	First grammar-based approach with T5 on Spider
PICARD (Scholak et al., 2021)	Transformer	Cross-domain	ACCLF: 71% ACCEX: 75%	Yes (T5)	Fine-tune T5 on Spider and introduce PICARD for semantically correct SQL
UnifiedSKG (Xie et al., 2022)	Transformer	Single (In this work, the focus is on WikiSQL)	-Using T5-base- ACCLF: 82.63% -Using T5-3b- ACCLF: 85.96%	Yes (T5)	Benchmarking T5 on Text-to-SQL

**ACCLF**, **ACCEX**, and **ACCASM** indicate the logical form accuracy, the execution accuracy, and approximate string-matching respectively.

EG stands for Execution guided.

Existing text-to-SQL models have not been fully embraced in the healthcare domain. Wang et al. (2020) stated that Text-to-SQL for EMRs was still under-explored. Based on literature review, only MedTS and TRESQ were introduced to assist medical staff with databases. Encouraged by previous success in the improvements of Text-to-SQL with transfer learning of the T5 model, this research aims to utilize transfer learning by fine-tuning the T5 model to develop a Text-to-SQL conversion model on EMRs and evaluate its performance. To the best of our knowledge, no existing work has fine-tuned the T5 model in Text-to-SQL for the healthcare domain. Furthermore, this study uses the WikiSQL dataset to benchmark the intended model against other models, in which WikiSQL was used, for performance comparison.

## 3 Research methodology

This research leverages the MIMICSQL dataset (Wang et al., 2020), the first publicly available dataset designed for Text-to-SQL tasks in the healthcare sector, to train and evaluate the MedT5SQL model.

We adopted the Cross-Industry Standard Process for Data Mining (CRISP-DM) methodology (Ncr and Clinton, 1999) to guide our research process. CRISP-DM is a widely used, structured approach for data mining projects, encompassing six key stages: business understanding, data understanding, data preparation, modeling, evaluation, and deployment (Marbán et al., 2009). This methodology has been successfully applied in various domains, including healthcare (Martínez-Plumed et al., 2021; Marshan et al., 2021).

To implement the MedT5SQL model, we utilized the Python programming language along with the PyTorch and HuggingFace Transformers libraries (Paszke et al., 2019; Huggingface.co, 2022) on the Google Colaboratory platform. Google Colab's provided GPU resources accelerated the computationally intensive deep learning processes involved in model training and evaluation.

## 4 Data analysis and results

### 4.1 Clinical objectives definition (business understanding)

The primary aim of this work is to generate an SQL query from written questions in the healthcare domain by utilizing natural language processing (NLP) through deep learning. The review of the relevant literature has revealed that the current state-of-the-art for Text-to-SQL conversion is to employ deep learning approaches with encoder-decoder architecture to achieve the required conversion. With the rise of the transformer's encoder-decoder architecture, various language conversion models were developed to improve NLP tasks using the transformer's encoder. They present large neural networks operating under a pre-train-fine tune paradigm where they are pre-trained over a large text corpus for a generic task, such as understanding a language, and then fine-tune on specific downstream tasks, such as summarization. Pre-training and fine-tuning these models facilitate leveraging transfer learning to improve the accuracy of various NLP tasks, including Text-to-SQL.

Throughout the literature review, it was observed that with the growth of transfer learning through pre-trained language models, deep learning has achieved promising results in this field (Guo et al., 2019; Hwang et al., 2019; Lyu et al., 2020; Choi et al., 2021; Pan et al., 2021). To get the most out of the transformer's encoder-decoder architecture and explore the limits of transfer learning, Raffel et al. (2020) built the Text-to-Text transfer transformer (T5) model as a unified large language model for all NLP tasks. The T5 model operates as an encoder-decoder with position encoding, attention mechanism, and self-attention for modeling all source tokens at once while understanding each token based on the context of the words around it. In Text-to-SQL conversion, Shaw et al. (2020) showed that the T5 model is able to learn Text-to-SQL conversion and operate with promising results. Encouraged by this work, research has been conducted presenting significant improvements in both WikiSQL and Spider benchmarks (Scholak et al., 2021; Xie et al., 2022). Despite the wealth of research in the field of Text-to-SQL, however, only two studies have been conducted focusing on the healthcare domain, proposing TREQS and MedTS models (Wang et al., 2020; Pan et al., 2021). TREQS is an original model developed entirely by Wang, Shi and Reddy, and MedTS benefits from transfer learning using a pre-trained model encoder, allowing it to outperform TREQS.

Considering the findings from the literature review, this study utilizes deep learning for Text-to-SQL conversion in the healthcare domain to develop a Text-to-SQL model named MedT5SQL employing transfer learning of the T5 transformer model. To the best of our knowledge, this work establishes the first model employing T5 in the healthcare Text-to-SQL conversion. This work focuses on using supervised deep learning to train the model on a healthcare-related dataset to achieve high conversion accuracy. Furthermore, MedT5SQL is benchmarked on WikiSQL dataset to evaluate its performance between the two datasets.

### 4.2 EMR data exploration (data understanding)

In this research we use MIMICSQL dataset that is created by Wang et al. (2020), to train and evaluate the MedT5SQL model. MIMICSQL is the first dataset created for Text-to-SQL tasks in the healthcare field. It is a large-scale dataset with 10,000 question-SQL pairs collected based on the Medical Information Mart for Intensive Care III (MIMIC III) dataset (Johnson et al., 2016). The medical information from MIMIC III was grouped into five tables for MIMICSQL as: demographics (Demo), laboratory tests (Lab), diagnosis (Diag), procedures (Pro) and prescriptions (Pres) (See Table 4 for information regarding MIMICSQL dataset). The question-SQL pairs were carefully constructed based on these tables. The pairs include questions to retrieve patient information directly from the database and reasoning questions to collect patient information from multiple tables. The pairs are divided into template questions (machine-generated) and natural language questions (human-annotated).

**Table 4:** Statistical summary of MIMICSQL dataset.

**Data**	**Stats**
Number of patients	46,520
Number of tables	5
Number of columns per table	Demo: 23, Diag: 5, Pro: 5, Pres: 7, and Lab: 9
Number of question-SQL pairs	10,000
Average template question length (in words)	18.39
Average natural language question length (in words)	16.45
Average SQL query length	21.14

**Figure 5:**
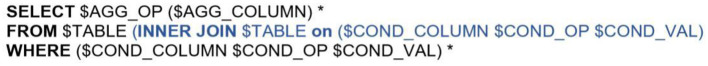
MIMICSQL SQL query structure.

The general structure of the SQL queries adopted in MIMICSQL is shown in Figure 5 and described as following:

- The SELECT clause allows multiple columns.
- The aggregation operators (AGG_OP) vary between NULL, MAX, MIN, COUNT and AVG.
- The column headers in the tables represent the question topic; therefore, AGG_COLUMN holds the question topic to retrieve the required information.
- The queries either retrieve the data from a single table or a new table generated from joining multiple tables through INNER JOIN by a condition.
- WHERE clause allows for one or multiple conditions.
- Only five condition operations (COND_OP) are considered in MIMICSQL, including =, >, <, >= and <=.

The WikiSQL dataset is used to benchmark MedT5SQL against other models that have used WikiSQL. This dataset contains 80,654 question-SQL pairs and it is larger than MIMICSQL with similar SQL structure.

### 4.3 Data acquisition and pre-processing

#### 4.3.1 Data acquisition

MIMICSQL was downloaded from Wang and Shi's (2020) repository on GitHub. They uploaded MIMICSQL in three separate files as data partitioning of the dataset, in the ratio of 0.8:0.1:0.1 for training, validation and test sets, respectively. In this work, we adopt the same data partitioning, using 8,000 pairs for training, 1,000 pairs for validation, and 1,000 pairs for testing the MedT5SQL model. The sets were stored on GitHub in the form of JSON files, and we extracted them into Pandas dataframes for easier manipulation. Similarly, WikiSQL is partitioned into three sets collected from the Hugging Face dataset library.

#### 4.3.2 Feature selection

The features relevant to this research in MIMICSQL dataset are (question_refine) and (sql), which represent the question-SQL pairs. Therefore, they were extracted for the training, validation and test datasets used in this research. The (question_refine) presents the (source_text) for the model, while (sql) presents the (target_text). In WikiSQL dataset, the question-SQL pairs are presented by (question) and (sql) features, renamed (source_text) and (target_text). However, this (target_text) was found to be a dictionary object where its entry (human_readable) presents the text form of the SQL, and thus, the SQL was extracted to form the (target_text).

#### 4.3.3 Handling missing and duplicate records

The datasets are inspected for missing data or duplicate pairs. In addition, the structure of the question-SQL pairs was inspected by checking random records to detect irrelevant records. No issues were identified in MIMICSQL, while WikiSQL had 189 duplicate pairs in the training set, 42 in the test set, and 29 in the validation set. These pairs were deleted before feeding the model with the data for the purpose of maintaining accuracy and avoiding biased performance.

#### 4.3.4 Tokenization

Prior to fine-tuning the T5 model, the source and target sequences were tokenized by splitting each text into its list of tokens (words) to understand the context. For the testing process, only the source text was tokenized before using it to generate the equivalent SQL query for model evaluation. A pre-trained T5Tokenizer from the T5ForConditionalGeneration module in the Hugging Face transformer package was used in this step. After number of experiments, the maximum number of tokens we were able to use for the source and target texts and train the MedT5SQL model are 150 tokens (original question) and 256 tokens (SQL Query), respectively. The pre-trained tokenizer not only splits the text into tokens but also converts the tokens into numeric representations to prepare the data before feeding it to the transformer-based deep neural model (Marshan et al., 2023). The tokenizer also adds padding tokens, which are used to fill the source and target text with extra tokens to standardize the number of tokens in each as required by deep neural models. Padding tokens also include guidance tokens that indicate the start and end of each text. As a result, the tokenizer results in “input_ids” and “attention_masks” fields, where the “input_ids” presents the list of tokens' IDs given to the model and “attention_masks” is a value of 0 or 1 mapped to each token, enabling the model to ignore padding tokens: 0 = masked/ignore and 1 = not masked.

#### 4.3.5 Data loader

In order to accelerate the training, validation and testing processes, PyTorch DataLoader was used to create data loaders for the tokenized datasets. Data loaders make it easier to manage the data and simplify the deep learning pipeline. They navigate the dataset by synchronously loading multiple batches of data using background processes called workers. Batches present the number of data samples run by the model in each training, validation, or test epoch. An epoch presents a complete pass of the whole dataset through the model. Following the work done by Pan et al. (2021) on MIMICSQL dataset, we used eight data samples per batch. The number of workers is set to four to allow faster data loading. To make the model more robust and avoid overfitting, shuffling was enabled for the training data loader to shuffle the data in every training epoch.

### 4.4 Modeling: developing the MedT5SQL model

Shaw et al. (2020) and Scholak et al. (2021) have showed that a pre-trained T5 model, especially the T5-base and T5-3b models have shown promising results as the current state-of-the-art for Text-to-SQL conversion. Motivated by these papers, this study developed the MedT5SQL model as a fine-tuned T5 model for text-to-SQL conversion in the healthcare domain. The MedT5SQL model went through several iterations until the successful model was achieved, as explained below. However, they all used the same model configurations.

#### 4.4.1 Model configuration and development environment

This study uses the T5ForConditionalGeneration module in the huggingface package to load the pre-trained T5-base model with its weights and operative configurations for fine-tuning. A list of the most important configurations related to the T5 architecture can be found in Table 5.

**Table 5:** T5-base configurations from hugging face transformers package.

**Configuration**	**Description**	**Value**
vocab_size	The number of different tokens represented by ‘inputs_ids' passed to the model	32,128
d_model	Encoder layers and the pooler layer size	768
num_layers	Number of encoder's hidden layers	12
feed_forward_proj	The activation function in the encoder	Relu
num_decoder_layers	Number of decoder's hidden layers	12
dropout_rate	Dropout rate for regularization	0.1
transformers_version	The version of transformers package	4.20.1
num_beams	Transformers use greedy decoding to select tokens with the highest probability	4
early_stopping	Use early stopping for regularization	True

MedT5SQL is trained on the Tesla P100 GPU from NVIDIA Corp offered by Google Colab and we used the parameters settings presented in Table 6. MedT5SQL sets the training and validation batch sizes to 8, similar to Pan et al. (2021) on MIMICSQL and the learning rate to 1e-4 as used by Shaw et al. (2020) and Scholak et al. (2021) for fine-tuning T5. The MedT5SQL model was trained using three different Epochs numbers 10, 15, 20, 50 and 100 to study its effect on the model performance. The validation was done using 15 epochs. The remaining parameters follow Pytorch-lightning (2022).

**Table 6:** MedT5SQL model configurations.

**Parameter**	**Value**
MODEL	t5-3b
TRAIN_BATCH_SIZE	8
VALID_BATCH_SIZE	8
TRAIN_EPOCHS	10, 15, 20, 50 and 100
VAL_EPOCHS	15
LEARNING_RATE	1e-4
MAX_SOURCE_TEXT_LENGTH	150
MAX_TARGET_TEXT_LENGTH	256
SEED	42
adam_epsilon	1e-8
weight_decay	0.0
n_gpu	1
gradient_accumulation_steps	16
warmup_steps	0
fp_16	False
output_dir	“/content/drive/MyDrive/MedT5SQL”
opt_level	apex
max_grad_norm	1.0

#### 4.4.2 MedT5SQL model

Initially, we attempted to fine-tune the Hugging Face T5-base model directly using PyTorch. However, despite successful training, the model failed to generalize to the Text-to-SQL task during testing, simply reproducing the input question instead of generating the corresponding SQL query. This indicated that the model had not adequately learned the translation task, likely due to insufficient task-specific guidance during fine-tuning.

**Figure 6:**
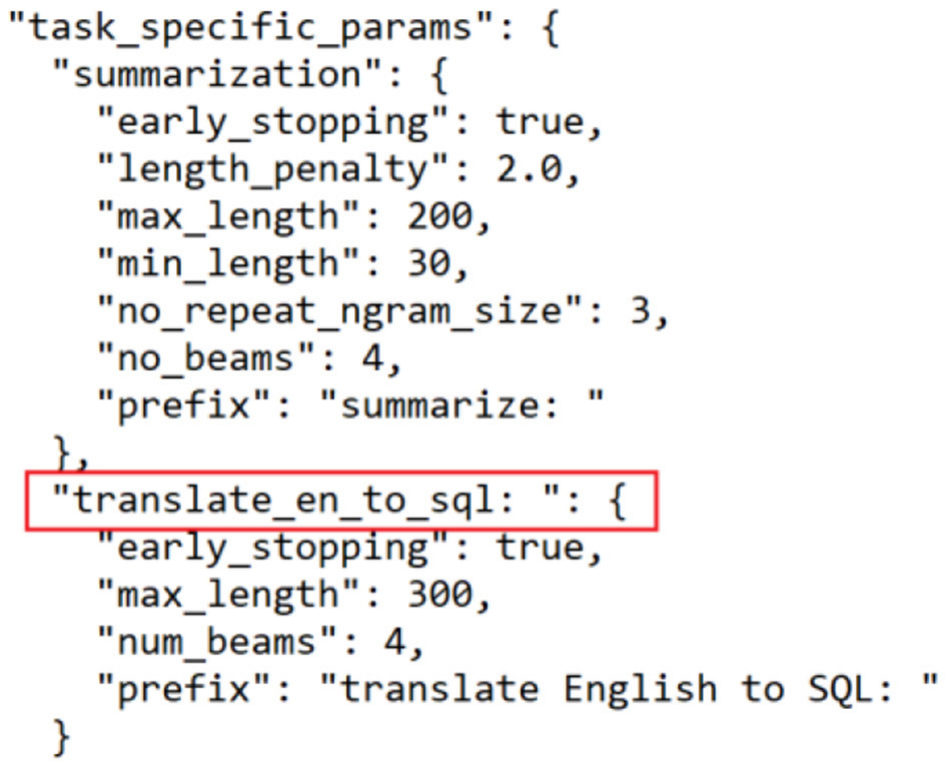
Modification on T5-base configuration model.

To address this, we incorporated a task-specific prefix (“translate English to SQL”) into the input sequence. This prefix acts as an explicit instruction to the model, prompting it to interpret the input as a Text-to-SQL translation problem. Additionally, we modified the T5-base model's configuration file to include parameters that reinforce the desired task (see Figure 6). These modifications guided the model's learning process and significantly improved its ability to generate correct SQL queries in response to natural language questions.

Nonetheless, training the T5 model with the new configuration file prevented it from using the weights of the pre-trained T5-base, and thus, the model functioned as a new model and not as a transfer learning of the T5 model, and with 8,000 training data, it achieved poor accuracy of <1% despite changing parameters, including training epochs, the optimizer, and the learning rate. Thus, as alluded to by Raffel et al. (2020) it is concluded that the T5 model should be able to understand the translation task without specifying the prefix.

To ensure that all the proper fine-tuning steps are performed, we developed the MedT5SQL model by utilizing the PyTorch Lightning framework (Lightning, 2022), which organizes and facilitates the process of building a model by abstracting the details of the training. It has a good Graphics Processing Unit (GPU) utilization and makes deep learning models flexible and easier to reproduce (Sawarkar, 2022). Using the Lightning Framework to build and train deep learning models requires the configuration of a LightningModule and Trainer parameters. LightningModule is used to structure the intended module to specify its behavior with each batch of training and validation data. Trainer uses the LightningModule with a specified dataset to automate the training and validation processes for the intended module.

#### 4.4.3 Lightning module configuration

In more details, to fine-tune the T5-base model using the Lightning Framework, MedT5SQL uses LightningModule to structure its implementation into four sections: initialization, training loop, validation loop and optimizer configuration (Pytorch-lightning, 2022). The LightningModule contains a function for each section to easily adopt any deep learning model to automate the training and validation loops with all the required components, such as epochs and optimizers. Overriding each of its functions allows MedT5SQL to specify its behavior in the training and validation to fine-tune the T5 model as required. The LightningModule for MedT5SQL was created and initialized given the model parameters listed in Table 6 and shown in Figure A1 in the Supplementary material.

#### 4.4.4 Training loop configuration

To activate the training loop of Lightning Framework for the fine-tuned T5-base model, MedT5SQL overrides the training functions of the LightningModule: *training_step* and *training_epoch_end*, as shown in Figure A2 in the Supplementary material. This loop is performed on the training dataset, loaded as batches by the data loader, to fine-tune the T5 model and obtains the training loss value using the *training_step* and *_step* functions as displayed in Figures A2, A3 in the Supplementary material. In T5 training, the T5 model's encoder uses the source tokens' IDs and masked values as input, while the decoder takes the encoder's output along with the target tokens' IDs (labels) to compute the training loss. The T5 model uses the cross-entropy loss function to compute the loss value required to modify the model's parameters during training (Raffel et al., 2020). The function *training_epoch_end* returns the average loss value of each training epoch. Lower loss values indicate a well-trained model.

#### 4.4.5 Validation loop configuration

To activate the validation loop of the Lightning Framework, MedT5SQL overrides the validation functions of LightningModule, as presented in Figure A4 in the Supplementary material. This loop uses the validation dataset, loaded as batches by the data loader, in the method ***_****step* to validate the model and obtain the validation loss (see Figure A3 in the Supplementary material). The ***_****step* method allows the model to generate the target text using the source text and evaluate the performance by comparing the generated query against the expected query and calculate the loss value. The function *validation_epoch_end* returns the average loss value of each validation epoch.

#### 4.4.6 Optimizer configuration

MedT5SQL is trained using Adafactor optimizer, the same optimizer used to pre-train the T5 model by Raffel et al. (2020) and other Text-to-SQL models built using the T5 model (Shaw et al., 2020; Scholak et al., 2021). The optimizer configuration is shown in Figure A5 in the Supplementary material. Moreover, we used AdamW optimizer, created by Loshchilov and Hutter (2017) to compare its performance against that of Adafactor (see Figure A6 in the Supplementary material).

#### 4.4.7 Trainer configuration

To develop MedT5SQL, PyTorch Lightning Trainer we automate the training and validation loops as presented in Figure A7 in the Supplementary material. The trainer was first created with the required arguments for the training process, such as the number of epochs, and was then given an object of MedT5SQL LightningModule class that contained the training and validation DataLoaders and loops. The developed MedT5SQL model presents a structured version of the first model we have developed using the Lightning framework, yet, without the use of a task-specific prefix, since the fine-tuning process was performed and organized successfully by the Lightning Framework.

### 4.5 Evaluation

To evaluate MedT5SQL model performance, the test dataset was used to assess the model on unseen data. The source text was tokenized and loaded in a DataLoader to feed MedT5SQL with natural language questions to generate equivalent SQL queries, as seen in Figure A8 in the Supplementary material. To generate the target text given the source text, the function “generate()” from the module T5ForConditionalGeneration is used. At the end, the tokenizer decodes the generated tokens into string form to output the SQL query sequence. This generated query was evaluated against the test dataset's target text to measure MedT5SQL performance.

MedT5SQL's performance was evaluated using logical form accuracy, known as exact match, and manual evaluation, in line with previous papers (Hwang et al., 2019; Wang et al., 2020; Pan et al., 2021). Additionally, we used approximate string matching to evaluate how close the MedT5SQL predicted query is to the expected query. The performance evaluation for MedT5SQL is presented in Figure A9 in the Supplementary material.

The manual evaluation was conducted by an independent reviewer with expertise in the medical domain. The reviewer was presented with a random sample of generated SQL queries paired with their corresponding expected queries from the test dataset. They assessed each generated query's correctness based on the following criteria:

- **Correctness:** Does the generated query accurately reflect the intended meaning and structure of the expected query?
- **Completeness:** Does the generated query include all necessary clauses and conditions?
- **Syntax:** Is the generated query syntactically valid?
- **Functional Equivalence:** If there are minor differences, does the generated query produce the same result as the expected query when executed on the database?

The reviewer assigned a score of “correct,” “partially correct,” or “incorrect” to each query. The manual evaluation score reported in our results represents the percentage of queries deemed “correct.”

A breakdown of logical form accuracy was performed on each SQL clause for further inspection. Figure A10 in the Supplementary material presents the evaluation process for the *SELECT* clause. MedT5SQL performance was evaluated in terms of the number of training epochs, as well as the optimizers, AdamW and Adafactor. It was also benchmarked against MIMICSQL and WikiSQL to examine its performance on different datasets.

## 5 Results and discussion

In this research, a Text-to-SQL conversion model named MedT5SQL was developed as the first fine-tuned T5-base model in the healthcare domain. The model was developed using MIMICSQL, a healthcare Text-to-SQL dataset. This section discusses the results of model evaluation and outlines its findings.

### 5.1 Performance evaluation on different training epochs and different optimizers

To understand the contribution of the number of training epochs to the performance of the model, MedT5SQL was trained on three different numbers of epochs: 10, 15, 20, 50 and 100. The performance was evaluated through accuracy measurement by comparing the generated SQL query against the expected SQL query using the test dataset (Hwang et al., 2019; Wang et al., 2020; Pan et al., 2021). The results are presented in Table 7, which shows that the accuracy measurements of MedT5SQL performance increased with the increasing number of training epochs.

**Table 7:** MedT5SQL performance evaluation using different parameter.

**# Training Epoc**	**AdamW Optimizer**			**Adafactor Optimizer**		
	**ACC**_LF_	**ACC**_ASM_	**ACC**_Manual_	**ACC**_LF_	**ACC**_ASM_	**ACC**_Manual_
10 epochs	57.9 %	97.455%	60%, 12 out of 20	58%	97.369%	65%, 13 out of 20
15 epochs	61.3%	97.716%	65%, 13 out of 20	62.6%	98.054%	75%, 15 out of 20
20 epochs	61.1%	97.81%	75%, 15 out of 20	63.1%	98.1%	80%, 16 out of 20
50 epochs	63.2%	97.926%	80%, 16 out of 20	68.9%	98.572%	85%, 17 out of 20
100 epochs	66.7%	98.016%	90%, 18 out of 20	80.63%	98.937%	90%, 18 out of 20

ACC_LF_, logical form accuracy by exact string matching; ACC_ASM_, approximate string-matching accuracy. ACC_manual_, manual evaluation derived by randomly examining 20 generated SQL queries.

To select the most efficient optimizer, the MedT5SQL model was developed using two different optimizers, Adafactor and AdamW, one at a time, and their performance was compared, as presented in Table 7. According to the analysis, Adafactor was more efficient for MedT5SQL, since it allowed the model to achieve higher accuracy compared to AdamW. Only when trained on 10 epochs did the model achieve 97.455% ACC_ASM_ with AdamW, compared to 97.369% with Adafactor. Nevertheless, it is worth noting that ACC_LF_ dropped by 0.2% when trained with AdamW on 20 epochs, compared to 15 epochs which could be a result of overfitting. ACC_LF_ rose by 0.5 under the same conditions using Adafactor. In general, Adafactor elevated MedT5SQL performance by 0.1–2% ACC_LF_, 0.3% ACC_ASM_ and 5% ACC_manual_, compared to AdamW. MedT5SQL achieved its highest accuracy of 80.1% ACC_LF_, 98.937% ACC_ASM_, and 90% ACC_manual_ when trained using Adafactor on 100 epochs. The values of ACC_ASM_ were extremely high, indicating the high similarities between the generated and the expected queries. Therefore, a breakdown evaluation was conducted on the SQL clauses to understand the reasons behind the differences between the ACC_LF_ and ACC_ASM_ values.

### 5.2 Performance on each SQL clause

To further analyse the generated SQL and investigate ACC_ASM_ values, we calculate the logical form accuracy (ACC_LF_) for each clause and shows the results in Table 8. The results confirm that Adafactor is more efficient for the MedT5SQL model. On the best performance, the exact match between the generated and expected queries was 96.8% and 97.01% for the *SELECT* and *FROM* clauses, respectively, while achieving 68.6% on the *WHERE* clause, which indicates that the model suffers mostly when generating the *WHERE* clause.

**Table 8:** Break down logical form accuracy (ACCLF) of MedT5SQL.

**# Training Epoc**	**AdamW Optimizer**			**Adafactor Optimizer**		
	**ACC (SELECT)**	**ACC (FROM)**	**ACC (WHERE)**	**ACC (SELECT)**	**ACC (FROM)**	**ACC (WHERE)**
10 epochs	93.6 %	95.1%	63.2%	90.2%	95.9%	64.2%
15 epochs	95.4%	95.4%	64.9%	95%	96.2%	66.4%
20 epochs	93.1%	96.1%	65.5%	95.4%	96.6%	66.2%
50 epochs	93.9%	97.03%	67.9%	96.1%	96.8%	67.6%
100 epochs	94.8%	97.53%	72.1%	**96.8%**	**97.01%**	**68.6%**

Bold values highlight the best performance of the model.

As shown in Table 9, the reason for this is related to the condition's value and operator as found by the manual evaluation. This was also demonstrated by Pan et al.'s (2021) evaluation of the accuracy of each component of the SQL query using multiple models, which confirmed that the condition's operation and values had lower accuracy than other components.

**Table 9:** Manual evaluation of MedT5SQL with Adafactor on 20 Epoch.

**Generated SQL query**	**Expected SQL query**
SELECT COUNT (DISTINCT DEMOGRAPHIC.“SUBJECT_ID”) FROM DEMOGRAPHIC INNER JOIN LAB on DEMOGRAPHIC.HADM_ID = LAB.HADM_ID WHERE DEMOGRAPHIC.”AGE“ ”30“ AND LAB.”FLAG“ = ”abnormal“	SELECT COUNT (DISTINCT DEMOGRAPHIC.”SUBJECT_ID”) FROM DEMOGRAPHIC INNER JOIN LAB on DEMOGRAPHIC.HADM_ID = LAB.HADM_ID WHERE DEMOGRAPHIC.“AGE” <“30” AND LAB.“FLAG” = “abnormal”
SELECT COUNT (DISTINCT DEMOGRAPHIC.“SUBJECT_ID”) FROM DEMOGRAPHIC INNER JOIN PRESCRIPTIONS on DEMOGRAPHIC.HADM_ID = PRESCRIPTIONS.HADM_ID WHERE PRESCRIPTIONS.”DRUG“ = ”Capso Fungin“	SELECT COUNT (DISTINCT DEMOGRAPHIC.”SUBJECT_ID”) FROM DEMOGRAPHIC INNER JOIN PRESCRIPTIONS on DEMOGRAPHIC.HADM_ID = PRESCRIPTIONS.HADM_ID WHERE PRESCRIPTIONS.“DRUG” = “Caspofungin”
SELECT COUNT (DISTINCT DEMOGRAPHIC.“SUBJECT_ID”) FROM DEMOGRAPHIC INNER JOIN LAB on DEMOGRAPHIC.HADM_ID = LAB.HADM_ID WHERE DEMOGRAPHIC.”DOB_YEAR“ ”2170“ AND LAB.”LABEL“ = ”Other Cells“	SELECT COUNT (DISTINCT DEMOGRAPHIC.”SUBJECT_ID”) FROM DEMOGRAPHIC INNER JOIN LAB on DEMOGRAPHIC.HADM_ID = LAB.HADM_ID WHERE DEMOGRAPHIC.“DOB_YEAR” <“2170” AND LAB.“LABEL” = “Other Cells”

Furthermore, the manual evaluation showed that the reasons behind the failed 3.2% ACC(SELECT) and 2.9% ACC(FROM) are related to column names and aggregators in the *SELECT* clause and the *INNER JOIN* or table names in the *FROM* clause as it can be noticed in Table 10. It was noted that a false *INNER JOIN* results in incorrect *WHERE* conditions.

**Table 10:** Evaluation of the SELECT and FROM clauses for MedT5SQL with Adafactor on 20 Epoch.

**Generated SQL query**	**Expected SQL query**	**Argument**
SELECT MAX (DEMOGRAPHIC.“AGE”) FROM DEMOGRAPHIC WHERE DEMOGRAPHIC.”MARITAL_STATUS“ = ”MARRIED“ AND DEMOGRAPHIC.”DOB_YEAR“ > ”2064“	SELECT COUNT (DISTINCT DEMOGRAPHIC.”SUBJECT_ID”) FROM DEMOGRAPHIC WHERE DEMOGRAPHIC.“MARITAL_STATUS” = “MARRIED” AND DEMOGRAPHIC.“DOB_YEAR” <“2064”	Failed SELECT clause: Incorrect aggregator and column name Failed WHERE clause: Incorrect operator
SELECT AVG (DEMOGRAPHIC.“AGE”) FROM DEMOGRAPHIC WHERE DEMOGRAPHIC.”ETHNICITY“ = ”WHITE“ AND DEMOGRAPHIC.”DIAGNOSIS“ = ”BRADYCARDIA“	SELECT COUNT (DISTINCT DEMOGRAPHIC.”SUBJECT_ID”) FROM DEMOGRAPHIC WHERE DEMOGRAPHIC.“ETHNICITY” = “WHITE” AND DEMOGRAPHIC.“DIAGNOSIS” = “BRADYCARDIA”	Failed SELECT clause: Incorrect aggregator and column name
SELECT COUNT (DISTINCT DEMOGRAPHIC.“SUBJECT_ID”) FROM DEMOGRAPHIC WHERE DEMOGRAPHIC.”DIAGNOSIS“ = ”ACIDOSIS“ AND DEMOGRAPHIC.”DAYS_STAY“ > ”7“	SELECT COUNT (DISTINCT DEMOGRAPHIC.”SUBJECT_ID”) FROM DEMOGRAPHIC INNER JOIN DIAGNOSES on DEMOGRAPHIC.HADM_ID = DIAGNOSES.HADM_ID WHERE DEMOGRAPHIC.“DAYS_STAY” > “7” AND DIAGNOSES.“SHORT_TITLE” = “Acidosis”	Failed FROM clause: Unidentified INNER JOIN Failed WHERE clause: Incorrect WHERE condition
SELECT COUNT (DISTINCT DEMOGRAPHIC.“SUBJECT_ID”) FROM DEMOGRAPHIC WHERE DEMOGRAPHIC.”DIAGNOSIS“ = ”SYNCOPE; COLLABORATION“ AND DEMOGRAPHIC.”ADMITYEAR“ ”2145“	SELECT COUNT (DISTINCT DEMOGRAPHIC.”SUBJECT_ID”) FROM DEMOGRAPHIC INNER JOIN DIAGNOSES on DEMOGRAPHIC.HADM_ID = DIAGNOSES.HADM_ID WHERE DEMOGRAPHIC.“ADMITYEAR” **<** “2145” AND DIAGNOSES.“SHORT_TITLE” = “Syncope and collapse”	Failed FROM clause: Unidentified INNER JOIN Failed WHERE clause: Incorrect WHERE condition and operator
SELECT DEMOGRAPHIC.“DIAGNOSIS”, PROCEDURES.”SHORT_TITLE“ FROM DEMOGRAPHIC INNER JOIN PROCEDURES on DEMOGRAPHIC.HADM_ID = PROCEDURES.HADM_ID WHERE DEMOGRAPHIC.”NAME“ = ”Bruce Harris“	SELECT DEMOGRAPHIC.”DIAGNOSIS”, DIAGNOSES.“ICD9_CODE” FROM DEMOGRAPHIC INNER JOIN DIAGNOSES on DEMOGRAPHIC.HADM_ID = DIAGNOSES.HADM_ID WHERE DEMOGRAPHIC.“NAME” = “Bruce Harris”	Failed FROM clause: Incorrect table name resulting in incorrect INNER JOIN condition
SELECT COUNT (DISTINCT DEMOGRAPHIC.“SUBJECT_ID”) FROM DEMOGRAPHIC INNER JOIN PROCEDURES on DEMOGRAPHIC.HADM_ID = PROCEDURES.HADM_ID WHERE DEMOGRAPHIC.”AGE“ ”54“ AND PROCEDURES.”LONG_TITLE“ = ”Squamous cell carcinoma of oral tongue/sda“	SELECT COUNT (DISTINCT DEMOGRAPHIC.”SUBJECT_ID”) FROM DEMOGRAPHIC WHERE DEMOGRAPHIC.“DIAGNOSIS” = “SQUAMOUS CELL CARCINOMA ORAL TONGUE/SDA” AND DEMOGRAPHIC.“AGE” <“54”	Failed FROM clause: Incorrectly generating INNER JOIN Failed WHERE clause: Incorrect WHERE condition

### 5.3 Benchmarking MedT5SQL on two datasets

Based on findings from past research, MedT5SQL on MIMICSQL was developed using the **Adafactor** optimizer. MedT5SQL was benchmarked on the WikiSQL dataset, explained in Section 2.1, to compare the performance on different types of questions. Table 11 presents a performance comparison between MedT5SQL developed using WikiSQL and MedT5SQL developed using MIMICSQL. It was found that the MedT5SQL model performed better when fine-tuned on MIMICSQL. Using MIMICSQL, **ACC**_**LF**_ achieved 58% and 62.6% when trained on 10 and 15 epochs, respectively, compared to 43.63% and 44.2% when using WikiSQL on the same number of epochs. Similarly, the ACC_ASM_ values obtained using MIMICSQL were 3.2–3.7% higher than those attained using WikiSQL.

**Table 11:** Accuracy evaluation of benchmarking MedT5SQL on two datasets.

**# Training Epoc**	**WikiSQL**			**MIMICSQL**		
	**ACC**_LF_	**ACC**_ASM_	**Time consumed**	**ACC**_LF_	**ACC**_ASM_	**Time consumed**
10 epochs	43.63%	94.1%	13 h	58%	97.369%	1 h 20 min
15 epochs	44.2%	94.26%	17 h	62.6%	98.054%	2 h
20 epochs	Model did not run			63.1%	98.1%	3 h
50 epochs	Model did not run			68.9%	98.572%	7 h 25 min
100 epochs	Model did not run			80.63%	98.937%	13 h 40 min

The difference in size between the datasets could be a contributing factor to this difference in performance. MIMICSQL has 8,000 question-SQL pairs for training and 1000 pairs for validation, while WikiSQL has 56,166 pairs for training and 8,392 for validation. Therefore, WikiSQL may need more training epochs to achieve better accuracies. However, benchmarking MedT5SQL on WikiSQL required longer execution time due to its enormous size, as shown in Table 11. For 20, 50 and 100 training epochs, the MedT5SQL did not run when it is trained on WikiSQL due to resource limitations, as Google Colab kept crashing when using the WikiSQL dataset on more than 15 epochs due to GPU memory shortage.

On WikiSQL, Xie et al.'s (2022) logical form accuracy evaluation of the UnifiedSKG model, baselined on T5-base, was shown to be 82.63% when trained on epochs between 50 and 200. In this work, WikiSQL achieved 43.63% on 10 epochs and 44.2% on 15 epochs. With sufficient resources, MedT5SQL may achieve equivalent results to UnifiedSKG. On MIMICSQL, Table 12 presents a comparison of the logical form accuracy between the developed model MedT5SQL and MedTS, the state-of-the-art model of MIMICSQL proposed by Pan et al. (2021). MedT5SQL outperforms MedTS knowing that it relies entirely on transfer learning, which offers a simpler architecture. According to Scholak et al. (2021) and Shaw et al. (2020), however, using T5-3b instead of T5-base, which we used in this research, can further improve the performance by around 13.5%. In this project, our attempt to create MedT5SQL by refining the T5-3b model was unsuccessful. The experiment faced challenges due to limitations in resources, specifically when the GPU exhausted its memory while processing the T5-3b model. This setback can be attributed to the substantial size of the T5-3b model, which comprises 3 billion parameters, in contrast to the 220 million parameters in the T5-base model.

**Table 12:** MedT5SQL and MedTS performance comparison.

**Model**	**ACC_LF_**
MedTS, trained on 100 epochs	78.4%
MedT5SQL, trained on 100 epochs	80.63%

Recent advancements in Text-to-SQL models have shown significant promise in improving the accuracy and efficiency of natural language interfaces for databases. In particular, models like ChatGPT (Liu et al., 2023), RASAT (Qi et al., 2022), and RESDSQL (Li et al., 2023) have reported impressive performance on various benchmark datasets. These models leverage large-scale pre-training and fine-tuning techniques, often employing transformers-based architectures, to achieve state-of-the-art results. However, their performance on healthcare-specific tasks and datasets remains less explored.

In the context of healthcare Text-to-SQL, the TREQS method proposed by Wang et al. (2020) stands out due to its reported 85% accuracy on the MIMICSQL dataset. While this accuracy is higher than that achieved by our MedT5SQL model, it is important to note that TREQS employs a rule-based approach with domain-specific templates, which may limit its generalizability to new datasets or query types. In contrast, our MedT5SQL model, based on the T5 large language model, offers greater flexibility and potential for adaptation to different healthcare contexts.

### 5.4 Limitations and future work

An accurate Text-to-SQL conversion model (MedT5SQL) is successfully developed for the healthcare domain, with a promising performance of 80.63% using transfer learning of the T5-base model. We argue that employing a larger T5 variant such as T5-3B model may yield improved performance due to their increased capacity. Also, using higher number of epochs would result in superior performance compared to existing models. In this study, we opted for the T5-base model due to resource constraints. Also, our research aimed to establish the feasibility and effectiveness of fine-tuning the T5 architecture for the specific task of Text-to-SQL conversion in the healthcare domain. We viewed the T5-base model as a suitable starting point for this initial exploration, allowing us to assess the potential of this approach before committing to the resource-intensive fine-tuning of the T5-3B model. Additionally, leveraging transfer learning by pre-training the model on larger and more diverse datasets beyond MIMICSQL could further enhance its ability to generalize to a wider range of healthcare queries. In addition, incorporating domain-specific knowledge into the model's architecture or training process could be a promising direction. This could involve incorporating medical ontologies, semantic representations, or rules-based components to guide the model's understanding and generation of healthcare-related SQL queries. Furthermore, while we focused on question-SQL pairs in this study, future work could explore the model's ability to handle a wider range of SQL queries, including complex queries with multiple clauses and conditions. Expanding the scope of supported queries would make the MedT5SQL model even more versatile and valuable for real-world healthcare applications.

Our research acknowledges the dynamic nature of large language model (LLM) development. While the T5 model served as an effective foundation for our study, we recognize that its relative performance may have evolved since our initial experiments, potentially impacting its standing among other state-of-the-art models. In this work, our primary objective was to investigate the potential of fine-tuning the T5 model for the specific domain of healthcare. This targeted approach allowed us to thoroughly explore the unique challenges and opportunities presented by this domain, revealing insights that may not be as readily apparent in broader, comparative studies. We believe that this deep dive into domain-specific fine-tuning holds considerable value, regardless of the T5 model's shifting position in the broader LLM landscape.

While a direct comparison of our fine-tuned T5 model with other state-of-the-art, fine-tuned LLMs would undoubtedly offer valuable insights, such an undertaking was beyond the scope of this initial study due to limitations on time and resources. However, we acknowledge the importance of such a comparison and consider it a crucial direction for future research. In our ongoing work, we aim to broaden our investigation by conducting comparative analyses that include other fine-tuned LLMs, further elucidating the strengths and weaknesses of various approaches in the context of healthcare.

We also acknowledge that the MIMICSQL dataset, while valuable, may not fully represent the diversity of EMR data and clinical queries encountered in real-world healthcare settings. This could lead to the model underperforming or exhibiting biases when applied to different patient populations or healthcare institutions. Additionally, the T5 model, like other large language models, can inadvertently learn and perpetuate biases present in its vast pre-training corpus. These biases could manifest as discriminatory or inequitable behavior in generated SQL queries. To deal with these biases, expanding and diversifying the training data to include a wider range of EMR types and clinical scenarios can help mitigate data bias. Model bias, on the other hand, can be addressed by developing evaluation metrics specifically for assessing bias in generated SQL queries and continuously monitoring the model's performance for potential biases. Finally, we argue that exploring techniques for fine-tuning the model to explicitly reduce biases, such as incorporating fairness constraints or re-weighting training examples should be an important direction of future research.

## 6 Conclusion

In recent times, patient health data is stored digitally in electronic medical records (EMRs), which healthcare professionals use to access patients' historical health information or for clinical trials. The onset of the global COVID-19 pandemic in early 2020 overwhelmed hospitals with patients, straining healthcare workers due to a shortage of medical staff relative to the patient load (Birkmeyer et al., 2020; Kruizinga et al., 2021; Iness et al., 2022). This crisis underscored the significance of EMRs and underscored the necessity for a more efficient communication method (Dagliati et al., 2021). The critical need is for an interface that facilitates seamless interactions between end users and databases, specifically a system capable of generating SQL queries in response to human language inquiries.

To meet this requirement, natural language processing (NLP) for Text-to-SQL, which allows non-technical users to generate SQL queries to communicate with databases using natural language text conversion has emerged as a suitable solution. This research reviews existing research on Text-to-SQL conversion and proposes a Text-to-SQL conversion model for EMRs retrieval. In this work we employ Large Language Model (LLM), namely Text-to-Text Transfer Transformer (T5) model, a transformer-based pre-trained model for all text-based NLP tasks, to develop the Text-to-SQL model.

The proposed model was developed by fine-tunning the T5 model on MIMICSQL dataset, the first Text-to-SQL dataset for healthcare domain. The model was benchmarked on two optimizers, different training epochs, and two datasets to compare the performance: WikiSQL and MIMICSQL datasets. The model's performance was evaluated by comparing the generated query, in which the model was given a text, against the expected query of the text. The experiments showed that the model was able to achieve high accuracy in generating SQL queries from natural language questions, particularly for medical question-SQL pairs. Further, evaluations of the performance on each SQL clause have shown the model's efficiency in generating these specific query types. This research demonstrates the potential of fine-tuning the T5 model to achieve state-of-the-art results for generating SQL queries from natural language questions in the healthcare domain. While the model's current scope is focused on question-SQL pairs, it provides a solid foundation for future research to expand into more comprehensive SQL generation tasks.

This research demonstrates the potential of fine-tuning the T5 model to achieve state-of-the-art results for generating SQL queries from natural language questions in the healthcare domain. While the model's current scope is focused on question-SQL pairs, it provides a solid foundation for future research to expand into more comprehensive SQL generation tasks. The MedT5SQL model, while promising, represents a significant step toward empowering healthcare professionals with efficient and intuitive access to EMR data. Its potential real-world deployment in clinical settings could revolutionize how medical staff interact with patient information, enabling them to quickly retrieve relevant data for informed decision-making. However, practical considerations such as seamless integration with existing Electronic Health Record (EHR) systems, development of user-friendly interfaces, and ensuring data security and privacy are crucial for successful implementation. Additionally, addressing potential limitations of the model, such as its current focus on question-SQL pairs and the need to adapt to varying EMR schemas, will be essential to maximize its impact.

Moving forward, further research should focus on expanding the model's capabilities to encompass a broader range of SQL queries, thoroughly evaluating its performance in real-world clinical environments, and exploring its potential applications in areas such as clinical decision support and medical research. By addressing these challenges and opportunities, MedT5SQL has the potential to transform the way healthcare professionals leverage EMR data, ultimately improving patient care and clinical outcomes.

## Data availability statement

Publicly available datasets were analyzed in this study. This data can be found at: https://github.com/wangpinggl/TREQS/tree/master/mimicsql_data/mimicsql_natural_v2; https://huggingface.co/datasets/wikisql.

## Author contributions

AMa: Writing – review & editing, Writing – original draft, Validation, Supervision, Software, Resources, Project administration, Methodology, Investigation, Formal analysis, Conceptualization. AA: Writing – review & editing, Writing – original draft, Visualization, Validation, Software, Resources, Methodology, Investigation, Formal analysis, Data curation, Conceptualization. AI: Writing – review & editing, Validation, Methodology, Investigation, Conceptualization. DB: Writing – review & editing, Project administration, Investigation, Data curation, Conceptualization. AMo: Writing – review & editing, Validation, Methodology, Investigation, Formal analysis, Data curation. MA: Writing – review & editing, Methodology, Investigation, Formal analysis, Data curation.
