# Supplementary material for: MedT5SQL: a transformers-based large language model for text-to-SQL conversion in the healthcare domain
Source: Front Big Data. 2024 Jun 26;7:1371680. doi: 10.3389/fdata.2024.1371680 (PMC11233734; doi:10.3389/fdata.2024.1371680)
Supplement: Supplementary file 1 [file Data_Sheet_1.docx]

# **Appendix**

**Figure A1**: Configuring the LighteningModule for the MedT5SQL Model

**Figure A2:** MedT5SQL Training Process

**Figure A3:** Training and Validating of the MedT5SQL Model

**Figure A4:** MedT5SQL Validation Process

**Figure A5:** MedT5SQL Adafactor Optimizer


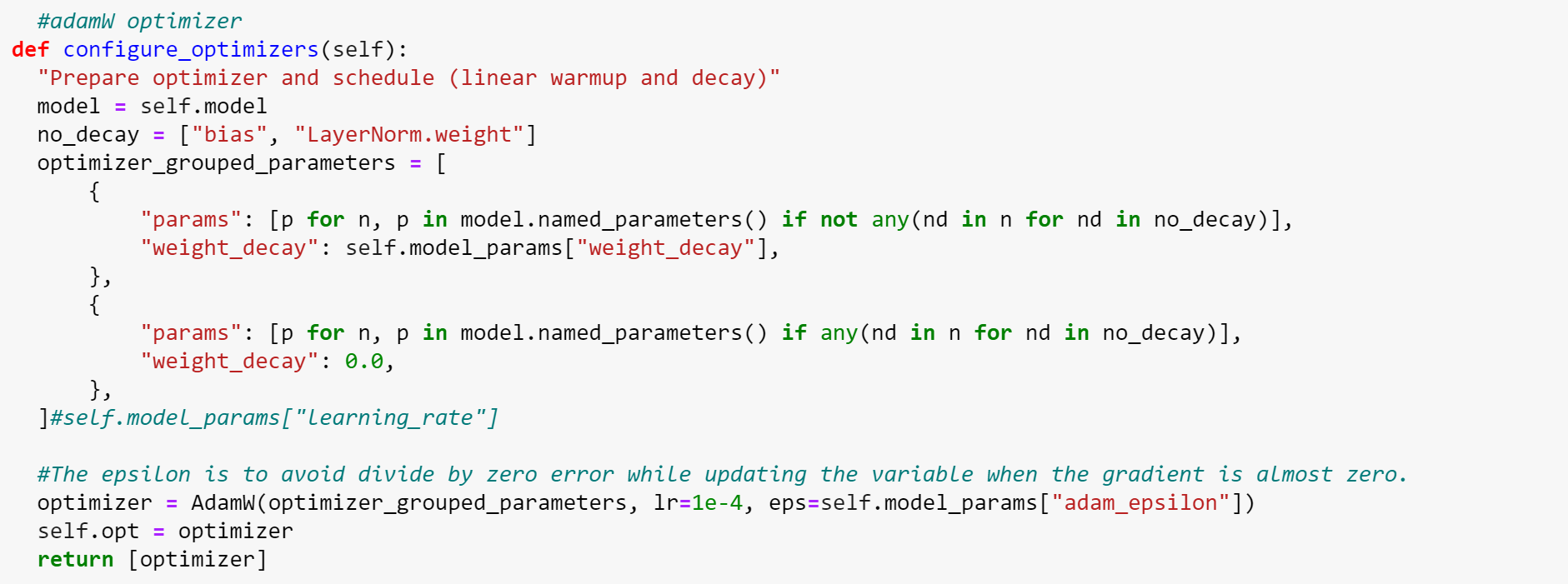


**Figure A6:** MedT5SQL AdamW Optimizer

**Figure A7:** MedT5SQL Trainer for using PyTorch Lightning Trainer


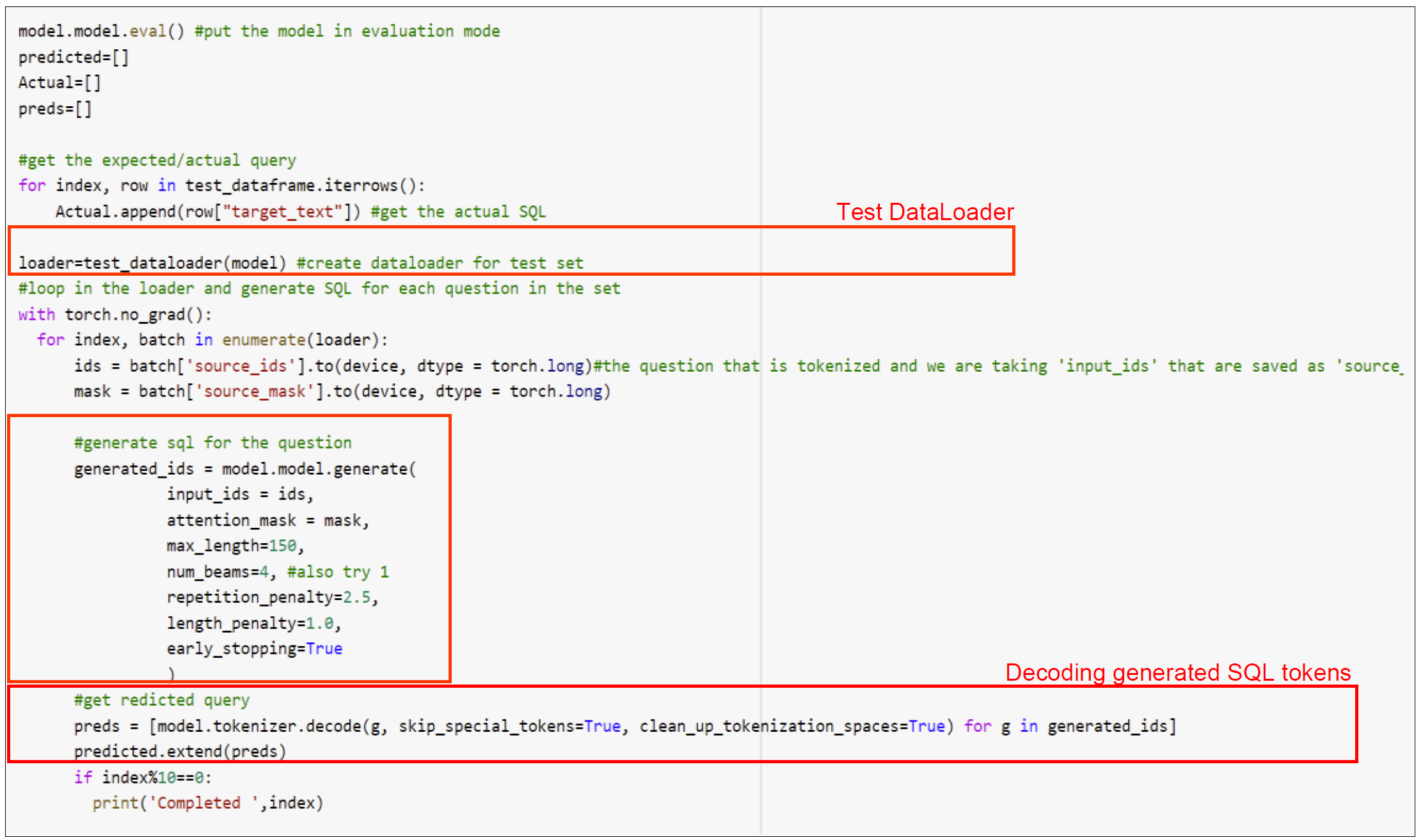


**Figure A8:** Testing MedT5SQL with the Test Dataset

**Figure A9:** MedT5SQL Accuracy Evaluation

**Figure A10:** Logical Form Accuracy for The SELECT Clause
